# Case report: characterization of a persistent, treatment-resistant, novel *Staphylococcus aureus* infection causing chronic mastitis in a Holstein dairy cow

**DOI:** 10.1186/s12917-020-02528-8

**Published:** 2020-09-15

**Authors:** Ellie J. Putz, Mitchell V. Palmer, Hao Ma, Eduardo Casas, Timothy A. Reinhardt, John D. Lippolis

**Affiliations:** 1 USDA-ARS-National Animal Disease Center, Infectious Bacterial Diseases Research Unit, Ames, IA USA; 2 USDA-ARS-National Animal Disease Center, Ruminant Disease and Immunology Research Unit, Ames, IA USA

**Keywords:** Mastitis, *Staphylococcus aureus*, Dairy, Myeloperoxidase, Antibiotic resistant, Chronic, Splendore-Hoeppli

## Abstract

**Background:**

Mastitis is the most common health concern plaguing the modern dairy cow and costs dairy producers estimates of two billion dollars annually. *Staphylococcus aureus* infections are prevalent, displaying varied disease presentation and markedly low cure rates. Neutrophils are considered the first line of defense against mastitis causing bacteria and are frequently targeted in the development of treatment and prevention technologies. We describe a case of naturally occurring, chronic mastitis in a Holstein cow (1428), caused by a novel strain of *S. aureus* that was not able to be cleared by antibiotic treatment.

**Case presentation:**

The infection was identified in a single quarter, 2 months into the cow’s first lactation. The infection persisted for the following 20 months, including through dry off, and a second calving and lactation. This case of mastitis was associated with a consistently high somatic cell count, however presented with no other clinical signs. This cow was unsuccessfully treated with antibiotics commonly used to treat mastitis, consisting of two rounds of treatment during lactation and an additional round at the beginning of dry off. The chronic infection was also unchanged through an experimental mid-lactation treatment with pegylated granulocyte-colony stimulating factor (PEG-gCSF) and an additional periparturient treatment with PEG-gCSF. We isolated milk neutrophils from 1428 and compared them to two cows challenged with experimental *S. aureus,* strain Newbould 305. Neutrophils from 1428’s milk had higher surface expression of myeloperoxidase compared to experimental Newbould challenged animals, as well as increased presence of Neutrophil Extracellular Traps. This suggests a heightened activation state of neutrophils sourced from 1428’s naturally occurring infection. Upon postmortem examination, the affected quarter revealed multifocal abscesses separated by fibrous connective tissues. Abscesses were most common in the gland cistern and collecting duct region. Microscopically, the inflammatory reaction was pyogranulomatous to granulomatous and consistent with botryomycosis. Colonies of Gram-positive cocci were found within the eosinophilic matrix of the Splendore-Hoeppli reaction within granulomas and intracellularly within the acinar epithelium.

**Conclusions:**

Collectively, we describe a unique case of chronic mastitis, the characterization of which provides valuable insight into the mechanics of *S. aureus* treatment resistance and immune escape.

## Background

Mastitis is estimated to cost the US dairy industry $2 billion per year [1]. One of the most common mastitis causing pathogens remains *Staphylococcus aureus* (*S. aureus*), which can appear in both chronic and acute varieties, with markedly low cure rates [2, 3]. *S. aureus* is known to escape immune clearance by adhering and infiltrating epithelial cells of the mammary gland which contributes to the difficulty to treat an infection [4, 5]. *S. aureus* can also be associated with walled-off aggregates seen histologically as Splendore-Hoeppli phenomena [6]. Strain specific phenotypes are also associated with *S. aureus* infections in cattle, including varying degrees of epithelial invasiveness, and inflammatory responses [5, 7, 8].

Neutrophils are a primary immune effector cell in response to an intramammary infection [9–11]. Circulating neutrophils express the cell adhesion molecule CD62L (L-selectin) on their cell surface. In response to an infection, local vascular signaling molecules interact with CD62L. This activation causes CD62L to be cleaved and shed from the cell surface, which facilitates cell migration into the tissue and helps target CD62L expressing immune cells, such as neutrophils, to the site of infection [10, 12–14]. An additional adhesion molecule, CD62E (E-selectin), is differentially expressed on vascular endothelial cells at the site of infection. Neutrophils have been shown to upregulate their surface expression of myeloperoxidase (MPO) in response to stimulus [15, 16]. Glycovariants of surface MPO are thought to bind to E-selectin [17] and maybe part of the mechanism that allows the movement of the neutrophils from the circulation into the mammary gland. Treatment of cows with PEGg-CSF can cause shedding of surface CD62L and up-regulation of cell surface MPO in neutrophils [18]. Experimental infection of the mammary gland has resulted in the appearance of neutrophils in the milk with the high surface level of CD62L and MPO, suggesting their translocation from the blood into the mammary gland of infected cows [18].

When they encounter a pathogen, neutrophils have multiple antimicrobial mechanistic weapons at their disposal. They can produce reactive oxygen species, phagocytose the bacteria, or eject their genomic material to capture the bacterium in what are called Neutrophil Extracellular Traps (NETs) that contain antimicrobial proteins [19–21]. The presence of neutrophil NETs in milk from infected cows can be observed by DNA stains of the milk fat. Of the three antimicrobial mechanisms employed by neutrophils, NETs have been shown to have a longer efficacy in milk than the others [20].

While antibiotics are the most common treatment for mastitis cases, alternative approaches do exist including preventative cytokine therapeutics such as pegylated granulocyte-colony stimulating factor (PEG-gCSF) (Imrestor/Pegbovigrastim, Elanco, IN USA). These alternative approaches have been shown to boost circulating neutrophil numbers, lower disease severity against mastitis challenge, and reduce the naturally occurring incidence of mastitis when administered during the periparturient period [22, 23].

## Case presentation

We describe the case of a three-year old Holstein dairy cow (1428) who presented with a naturally-occurring, subclinical mastitis infection in her left hindquarter, approximately two months into her first lactation. Milk samples from cows in our research herd are periodically monitored for bacterial growth and changes in SCC (Somatic Cell Count) to monitor animal and udder health. Additional samples are collected if naturally occurring mastitis is suspected or for various scientific uses. Cow 1428 was born and raised on the USDA campus within the Holstein research herd. Mastitis was observed at a routine daily milking and culture of the sample was performed. Milk from quarters of interest was aseptically collected, by hand milking, and SCC sample values were determined by Dairy Lab Services (IA, USA). For bacterial counts, aseptically collected milk samples were plated on Trypticase Soy Agar with 5% sheep blood plates (BD Biosciences, CA, USA Cat. No.221261), and incubated overnight at 37 °C, prior to colony counting. An isolated colony was typed by the Iowa State University Veterinary Diagnostic Laboratory (ISU VDL) and identified as *S. aureus*. The *S. aureus* strain was sequenced and designated as SA1428 [24]. The infection remained subclinical, with no identifiable drop in milk yield, no visual signs of inflammation including teat hardening, redness, or milk chunkiness, but was continuously identifiable by moderately increased SCC and bacterial culture. Multiple SCC and bacterial counts were determined over the course of several months. At the initial detection of the infection SCC in the infected quarter were 3.8 × 10^6^ cells/ mL with bacteria counts > 3000 cfu/mL. Other quarters had no detectable bacteria and SCC below 7.5 × 10^4^ cells/ mL. Cow 1428 was not isolated from the herd, however, no other cows became naturally infected with the novel *S. aureus* pathogen to our knowledge.

Cow 1428 was treated with antibiotics, daily for five days, with cephapirin sodium (ToDAY, Boehringer Ingelheim, MO, USA) twice daily, and additionally pirlimycin hydrochloride (PIRSUE, Zoetis, NJ, USA) once daily. In our herd, this treatment has been successful at clearing experimentally induced *S. aureus* infections (Newbould 305 strain). When antibiotics did not clear the infection (as confirmed by bacterial culture) an additional round of antibiotics was completed two months later, which also failed to clear 1428’s infection. Numerous rounds of antibiotic treatment may not be a common commercial practice, but was appropriate within our research herd where previously we have been able to clear experimental *S. aureus* infections with this specific treatment and where milk is not used for human consumption. Interestingly, susceptibility testing of SA1428 by the ISU VDL, revealed susceptibility to several antibiotics (Ampicillin, Ceftiofur, Cephalothin, Erythromycin, Oxacillin, Penicillin, Penicillin/Novobiocin, Pirlimycin, Sulfadimethoxine, and Tetracycline). Mid-lactation, cow 1428 was treated off-label with a cytokine-based, PEG-gCSF therapy (Imrestor/Pegbovigrastim, Elanco, IN, USA), which was administered in two subcutaneous doses of 2.7 mL of 15 mg PEG-gCSF 7 days apart. While on-label use is designed for periparturient administration, our group was interested if the PEG-gCSF targeted neutrophil expansion would have an effect on 1428’s chronic infection. After PEG-gCSF treatment, circulating blood neutrophils increased from 2× 10^9^ cells per liter of blood to 54 × 10^9^ cells per liter of blood at their peak, 2 days post the second PEG-gCSF injection. In her infected quarter, cow 1428’s SCC also increased, from 1.11 × 10^6^ cells per milliliter of milk to 5.17 × 10^6^ cells per milliliter of milk, peaking 4 days post the second PEG-gCSF injection. Despite the increased presence of circulating neutrophils and milk SCC, cow 1428 did not clear the *S. aureus* infection. After being bred and confirmed pregnant, cow 1428 was dried off approximately 60 days prior to calving. In accordance with general dry-off practice, she was treated with cephapirin benzathine (ToMORROW, Boehringer Ingelheim, MO, USA). Seven days prior to cow 1428’s calving date she was treated again with an injection of PEG-gCSF, and again on the day of calving, as directed by on-label use of the product. Despite this treatment, 1428’s *S. aureus* infection presented immediately with the start of her second lactation.

We sought to characterize the infected mammary gland environment to identify phenotypes associated with SA1428 infection as compared to experimentally infected *S. aureus*. For the experimentally infected samples, we utilized resident Holsteins five weeks post experimental infection with *S. aureus* Newbould 305. For another ongoing study on the USDA National Animal Disease Center campus, eight Holstein cows were challenged by intramammary infusion in a single quarter with 150 CFU of *S. aureus* (Newbould). Subclinical, chronic infections developed in all cows. Five weeks after challenge, the Newbould infected cow with consistently high SCC values, and the Newbould infected cow with consistently low SCC values had milk samples collected for comparison along with milk from 1428’s naturally occurring infection. Over three consecutive days, 1428’s SCC for her infected quarter averaged 3.02 ± 0.78 × 10^6^ cells per milliliter of milk, the high SCC cow averaged 12.59 ± 6.55 × 10^6^ cells per milliliter of milk, and the low SCC cow averaged 0.24 ± 0.08 × 10^6^ cells per milliliter of milk.

From *S. aureus* infected quarters 100–150 mL of milk was collected into 50 mL conical tubes. Samples were spun for 40 min, at 10,000 x g, at 4 °C to separate for pelleted milk cells for flow analysis and milk fat. Top milk fat layers were scraped into separate tubes, washed with PBS and protease inhibitor, and frozen for subsequent NET analysis.

**Fig. 1:**
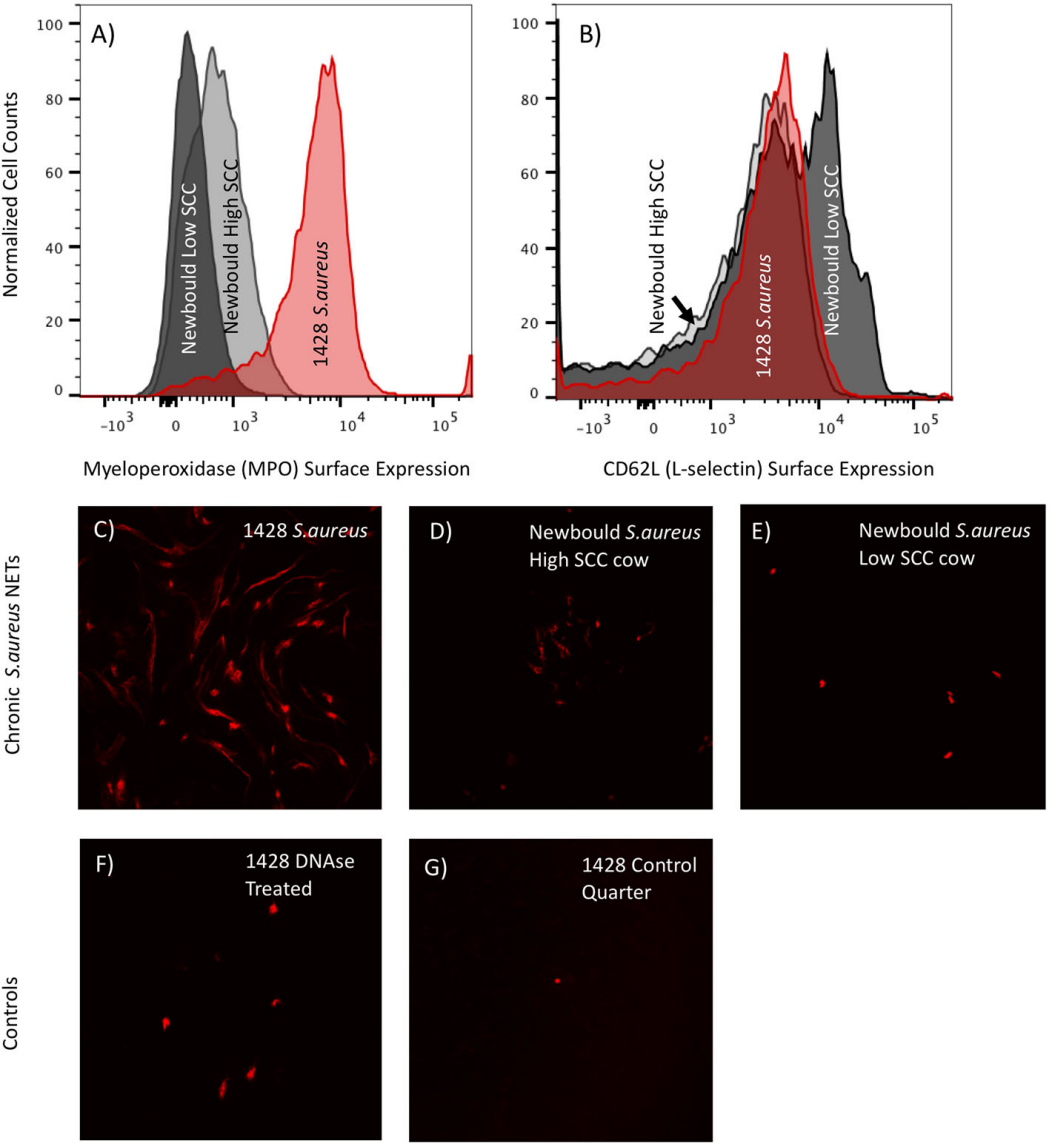
Flow cytometry and microscopy of NETs sourced from chronically *S. aureus* infected milk from 1428 and two Newbould infected cows (high and low somatic cell responders). Flow Cytometry of neutrophils isolated from infected milk was analyzed for surface expression of (**a**) myeloperoxidase and (**b**) CD62L (L-selectin). The red histogram depicts cell isolated from 1428 milk, dark gray is from a chronic Newbould challenged low SCC cow, and light gray represents a chronic Newbould challenged high SCC cow. Milk fat was additionally evaluated for the presence of Neutrophil Extracellular Traps (NETs). (**c**) 1428 *S. aureus* infected milk fat shows increased NET presence compared to both high (**d**) and low (**e**) SCC cows challenged with Newbould. Control NET staining shows DNAse treatment of 1428’s infected milk fat (**f**), and staining of clean healthy quarter milk fat from cow 1428

Milk from the centrifuged samples was poured off, and cell pellets were placed on ice and resuspended in 1 mL media (L-glutamine, 10% FBS supplemented complete RPMI). Cell suspensions were layered over density gradients (Histopaque 1077, Sigma Aldrich, MO, USA, Cat No. 10771-500ML) spun for 20 min at 1500 x g, and had buffy coats removed leaving a highly neutrophil enriched cell pellet. Cell pellets were washed once with PBS and live cell counts were determined by cell counter (TC20 automated cell counter, BioRad, CA, USA). We used flow cytometry to evaluate the surface expression of MPO and L-selectin on neutrophils sourced from milk from infected quarters. To compare MPO and L-selectin expression over a range of SCC, we sampled milk from the Newbould infected cows with the highest and lowest SCC to compare with milk from cow 1428. Live milk cells were washed and resuspended in flow buffer (BioLegend, CA, USA, Cat. No. 420201). Individual primary, secondary, and directly conjugated antibodies were added to cell suspensions and incubated at room temperature for 15 min in the dark, with a flow buffer wash step between each antibody set. Samples were run on a Becton Dickinson LSR II flow cytometer and all analyses were performed with FlowJo software (FlowJo LLC, Ashland, OR, USA). Neutrophil gating was determined by forward and side scatter. Live, singlet milk cells were gated for CD45 (Monoclonal Antibody Center, Washington State University, USA. Cat. No. BOV2039). CD45^+^ cells were separately assessed for MPO (BioRad, Hercules, CA, USA Cat. No. VPA00193) and CD62L (BioLegend, San Diego, CA, USA Cat. No. 304824) surface expression. Flow cytometry of milk derived neutrophils from the three cows showed that cow 1428 had the greatest MPO surface expression (Fig. 1a). Between the Newbould infected cows, the high SCC cow also showed higher surface MPO expression compared to the low SCC cow (Fig. 1a). Surface expression of L-selectin revealed comparable levels on 1428 and the high SCC Newbould infected cow, but both were reduced compared to the low SCC Newbould infected cow (Fig. 1b).

Neutrophils are known to produce NETs with activation which results in cell death. To capture information about NET-forming neutrophils, we stained milk fat for DNA as described previously [16], which is indicative of NET formation from all three cows. Microscopy showed that 1428 had the greatest NET presence (Fig. 1c), compared to both Newbould infected cows (Fig. 1d, e). DNAse treated and healthy milk fat controls are shown in Fig. 1f, g. Samples were analyzed via confocal microscopy imaging using a Nikon A1R+ laser scanning microscope and NIS-Elements imaging software. Slide images are shown at the 20X objective, 75 numerical aperture, as imaged using a GaASP detector, 561 laser.

**Fig. 2:**
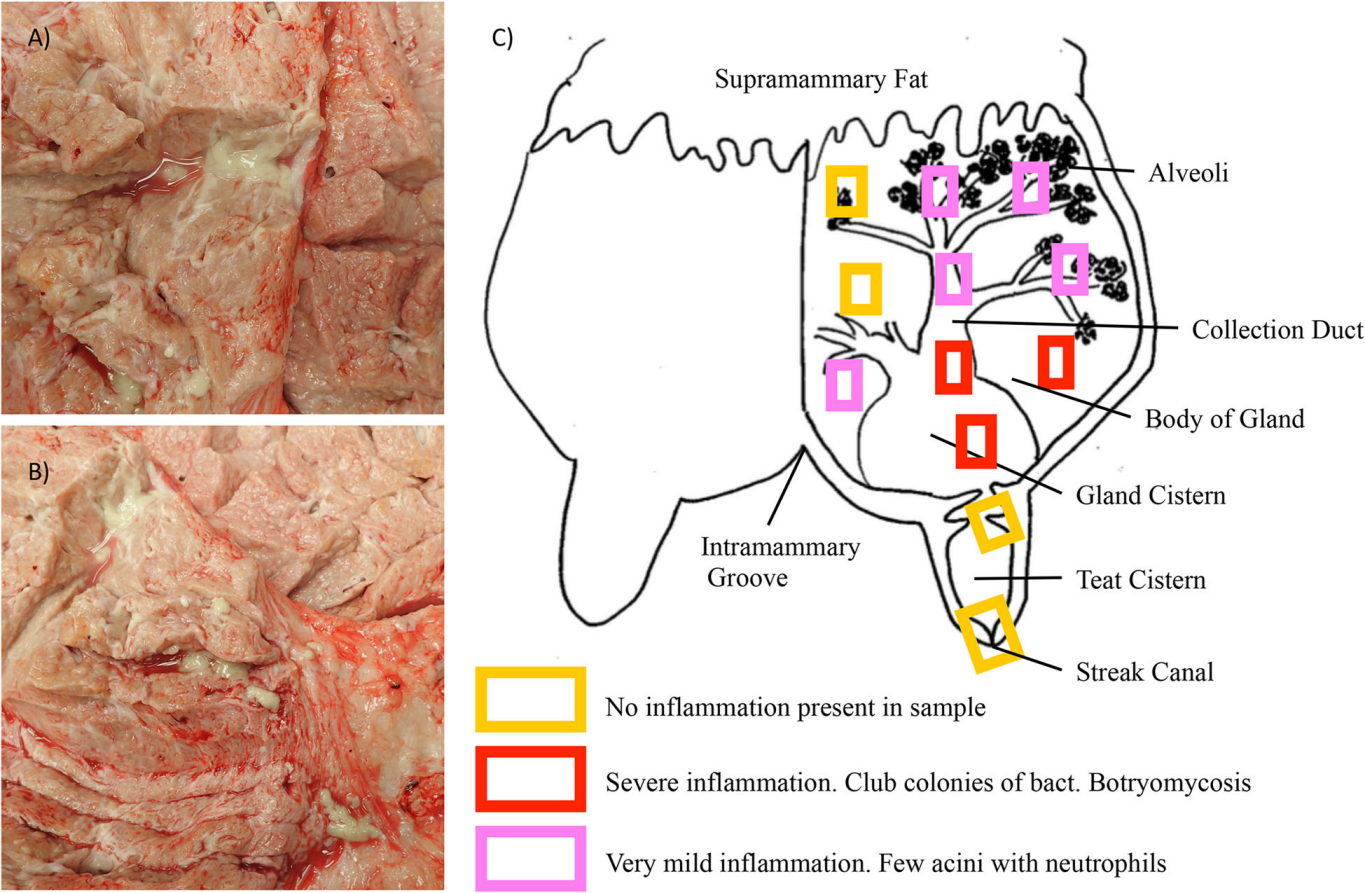
Gross pathology and necropsy collection outline detailing levels of inflammation. Gross pathology of 1428’s infected quarter (**a**, **b**). Note multifocal purulent exudate found in abscesses surrounded by fibrous connective tissue. The mammary gland was sampled as detailed in (**c**), capturing tissue from physiologically meaningful regions of the mammary gland. Also depicted, general levels of inflammation found at each section level (**c**). Diagram in (**c**) adapted from [25]

**Fig. 3:**
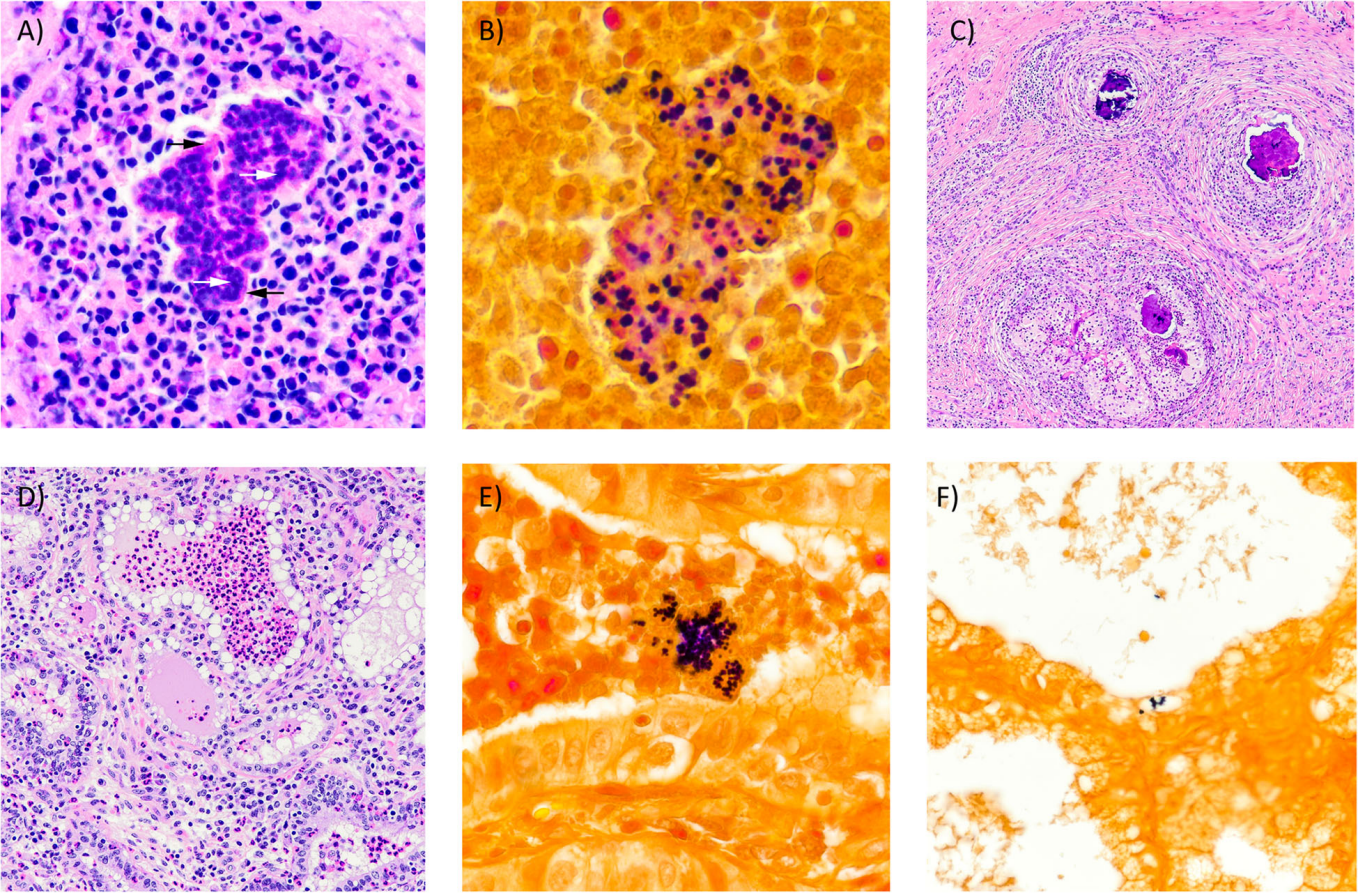
Photomicrographs of mastitic quarter from 1428. (A) Pyogranulomatous infiltrate. Note colonies of basophilic cocci (white arrows) within brightly eosinophilic and radiating matrix of Splendore-Hoeppli reaction (black arrows). H&E 20X. (B) Cocci within eosinophilic matrix are Gram-positive. Gram stain 40X. (C) Granulomas with abundant peripheral fibrosis and central areas of dystrophic mineralization. H&E 4X. (D) Acini containing numerous neutrophils. H&E 10X. (E) Intraluminal Gram-positive cocci within acinus. Gram stain 40X. (F) Intracellular Gram-positive cocci. Gram stain 20X

Cow 1428 was euthanized approximately 20 months after the first identification of infection by lethal injection of barbiturates by our institutional veterinarian. Gross pathology of the infected quarter of the mammary gland can be seen in Fig. 2a, b**.** The infected quarter was systematically sampled by obtaining samples from 12 different sites; 3 each from the proximal and distal gland body of the gland, 3 from the collecting duct region and 1 each from the gland cistern, teat cistern and streak canal as illustrated in Fig. 2c. Gross examination revealed multifocal abscesses and increased amounts of fibrous connective tissue, most notably in the collecting duct and gland cistern regions. Tissue samples (≤0.5 cm thick) were fixed by immersion in 10% neutral buffered formalin for 24 h, then transferred to 70% alcohol followed by standard paraffin embedding techniques. Paraffin embedded samples were cut to 4 μm thick sections, transferred to Superfrost Plus™ charged microscope slides (Thermo Fisher, MA, USA) and stained with hematoxylin and eosin (H&E). Adjacent sections were stained by the Hucker-Twort technique for visualization of Gram-positive and Gram-negative bacteria. Microscopically, samples from the teat sphincter and teat cistern were normal, with minimal if any inflammation (Fig. 2c) and no bacteria present, confirmed by Gram stain. Samples from the gland cistern and collecting duct regions contained multifocal suppurative to pyogranulomatous infiltrates. Some regions contained distinct infiltrates of only neutrophils surrounding colonies of Gram-positive cocci embedded in a brightly eosinophilic, homogenous matrix, which radiated outward; interpreted to be Splendore-Hoeppli reaction (Fig. 3a, b). In these same regions, there were also pyogranulomatous to granulomatous infiltrates arranged in nodules separated by prominent bands of fibrous connective tissue (Fig. 3c). These nodular infiltrates contained variable numbers of extracellular Gram-positive cocci. In the alveolar duct and body regions of the gland, numerous acini contained infiltrates of large numbers of neutrophils (Fig. 3d). In such acini, Gram-positive cocci were found both individually and in small colonies (Fig. 3e). Some glands were absent of inflammatory infiltrates, but one to several Gram-positive cocci could still be found adhered to or within epithelial cells (Fig. 3f).

To further document *S. aureus* strain SA1428, its genome was sequenced. The genome sequencing data have been deposited in NCBI Sequence Read Archive under accession number PRJNA609126. The de novo genome assembly is available at NCBI with the accession number CP048431-CP048432.

## Discussion and conclusions

This unique case report sheds light on important host and pathogen interactions that should be further investigated to be utilized in the development of mastitis treatment and preventative technologies. The presence of heightened immune cell activation in the mammary gland identified by present neutrophils, but lack of bacterial clearance, raises questions about the mechanisms of inflammation regulation and immune escape strategies of *S. aureus* as a mastitis causing pathogen. Low cure rates of *S. aureus* mastitis have been attributed to components of host and pathogen genetics, environmental exposure, and antibiotic resistance [26]. Additionally, while it’s known that *S. aureus* can evade neutrophil killing and gain intracellular access to epithelial cells [26], much more needs to be understood about why the immune response is ineffective and how *S. aureus* escape mechanisms function. In this work we demonstrate a correlation between infections caused by two strains of *S. aureus* and differences in surface expression of proteins of interest on milk neutrophils. Milk neutrophils sourced from cow 1428 had substantially increased surface expression of MPO (Fig. 1a), which has been associated with cell activation [16] as well as accumulation within mastitic mammary glands [18]. Also consistent with neutrophil activation, levels of surface CD62L were comparable between 1428 cells and the high SCC Newbould cow, but both appeared to have increased shedding of CD62L compared to the low SCC cow (Fig. 1b). These observations are consistent with the role of CD62L as an adhesion molecule important for the targeting cells to the site of infection. These observations are also supportive of the hypothesis that MPO expressed on the cell surface may be a ligand for E-selectin and potentially plays a role in cell migration to localized infections. Lastly, imaging showed more NETs present in 1428’s milk fat than in either of the Newbould challenged cows (Fig. 1c, d, e). Collectively these findings suggest that cow 1428 had heightened neutrophil activation within the mammary gland compared to cells from experimentally infected animals. It is also clear that this activation is not driven strictly by accumulated cell numbers as the high SCC Newbould infected cow had four-fold higher SCC numbers than 1428. Of interest, between the two Newbould experimentally-infected animals, the high SCC Newbould infected cow had higher surface expression of MPO compared to the low SCC Newbould infected cow (Fig. 1a), reduced surface expression of CD62L (Fig. 1b), and increased NET presence (Fig. 1d,e), which is supportive that these parameters are capturing biological activation. These findings should be further validated in the context of hypothesis driven experimental studies.

Many different strains of *S. aureus* exist that are capable of causing mastitis in dairy cattle. Variations in mastitis-causing strains include differences in the genotypic expression of virulence factors, biofilm production, cellular infiltration, and antimicrobial activity [27]. Comparison of an infection by SA1428 and SA Newbould highlights the phenotypic variation between strains that can be observed, both in terms of host immune cell response and response to antibiotic treatment; Newbould being successfully cleared and SA1428 persisting. The antibiotic susceptibility results of *S. aureus* SA1428, but failure to treat in vivo, suggests that the persistence of 1428’s infection may be contributed to physical escape by the bacteria. Histologically, inflammation of 1428’s infected quarter can be considered chronic in nature with pyogranulomatous and granulomatous lesions and increased fibrosis. The largest numbers of Gram-positive cocci were seen within the eosinophilic matrix of the Splendore-Hoeppli reaction associated with pyogranulomatous lesions and the nodular granulomatous infiltrates surrounded by large bands of fibrous connective tissue. Both settings provide protection from antibiotic treatment.

The naturally occurring, chronic case of *S. aureus* mastitis of cow 1428 describes the identification of strain SA1428, and describes an associated treatment-resistant phenotype. Our unique evaluation of activation levels of mammary sourced neutrophils, and detailed look at weakly characterized histology phenomena, contributes to the general knowledge of the behavior of chronic *S. aureus* infections, and offers several opportunities for hypothesis driven research to explore these findings.

## Data Availability

The genome sequencing data for SA1428 has been deposited in NCBI Sequence Read Archive (accession number PRJNA609126), and the de novo genome assembly is available at NCBI (accession number CP048431-CP048432). All additional datasets used and/or analyzed during the current study are available from the corresponding author on reasonable request.

## Abbreviations

SCC: Somatic Cell Count
MPO: Myeloperoxidase
PEG-gCSF: Pegylated granulocyte-colony stimulating factor
NET: Neutrophil extracellular trap
H&E: Hematoxylin and eosin
